# Adequacy of Hemodialysis and Its Associated Factors among Patients Undergoing Chronic Hemodialysis in Dar es Salaam, Tanzania

**DOI:** 10.1155/2020/9863065

**Published:** 2020-02-10

**Authors:** Samina S. Somji, Pascal Ruggajo, Sibtain Moledina

**Affiliations:** Department of Internal Medicine, Muhimbili University of Health and Allied Sciences, P.O. Box 65001, Dar es Salaam, Tanzania

## Abstract

The worldwide prevalence of maintenance hemodialysis continues to rise. An adequate delivery of hemodialysis dose as measured by *Kt*/*V* or urea reduction ratio is a crucial determinant of clinical outcome for chronic hemodialysis patients. The aim of this study was to assess the adequacy of hemodialysis and its associated factors among patients undergoing chronic hemodialysis in Dar es Salaam. This was a cross-sectional study done on patients undergoing chronic hemodialysis in four dialysis centers in Dar es Salaam. Sociodemographic information and treatment characteristics were collected. Urea reduction rate and single-pool *Kt*/*V* were calculated to determine the adequacy of hemodialysis. The data were analyzed and any associated factors for inadequate hemodialysis were determined using a chi-square test and a logistic regression analysis. A total of 143 patients participated in the study. Males represented 65.7% of the study population. The mean age (±SD) was 51.7 ± 1.2 years. Only 34.3% (based on urea reduction ratio (URR)) and 40.6% (based on *Kt*/*V*) of patients received adequate hemodialysis. The univariate analysis showed that males were more likely to have inadequate dialysis (65.6% versus 48.0%, *p*=0.048 based on *Kt*/*V*). Patients using hemodialyzers with dialyzer surface area less than 1.4 m^2^ received significantly less hemodialysis dose than those with more than 1.4 m^2^ (69.0% versus 41.2%, *p*=0.02, by URR) (62.7% versus 35.3%, *p*=0.03, by *Kt*/*V* criteria). Patients who had hemoglobin <10 g/dl received significantly inadequate hemodialysis dose as compared to patients with hemoglobin ≥10 g/dl by *Kt*/*V* criteria (69.8% versus 51.3%, *p*=0.03). None of the factors acquired significance in the multivariate analysis. The proportion of patients receiving an adequate hemodialysis dose is low (34.3% based on URR and 40.6% based on *Kt*/*V*). Male gender, dialyzer surface area of <1.4 m^2^, and hemoglobin level of <10 g/dl were associated with an inadequate delivered dose of hemodialysis in the univariate analysis but not in the multivariate analysis. This study can increase awareness about the importance of measuring hemodialysis adequacy and giving the correct hemodialysis dose to achieve the intended benefit.

## 1. Introduction

Chronic kidney disease (CKD) has nearly doubled as a cause of death worldwide between 1990 and 2010 and was the 18^th^ highest cause of death worldwide in 2010 [1]. It is estimated that by 2030 more than 70% of patients with end-stage renal disease will be living in low-income countries, such as those in Sub-Saharan Africa. In Kinshasa, the prevalence of CKD was found to be 12.4% among adults in the general population. In Uganda, 20% of HIV/AIDS patients had CKD [2, 3]. There are many potential causes of CKD in Sub-Saharan Africa which makes kidney disease burdensome in the region. In addition to noncommunicable diseases, communicable diseases such as infectious glomerulonephritis, schistosomiasis, leishmaniasis, and HIV infection are common and can cause CKD [4, 5].

Patients with end-stage renal disease (ESRD) are unable to sustain life without dialysis support. Hemodialysis is the transport process by which a solute passively diffuses down its concentration gradient from one fluid compartment (either blood or dialysate) into the other [6]. The goal of hemodialysis is exiting of the toxins from the body and preservation of its intracellular and extracellular composition in normal range as much as possible. The worldwide prevalence of long term dialysis continues to rise [7, 8] driven in part by strong trends towards the initiation of dialysis earlier in the natural history of CKD than was the practice previously [9].

The adequacy of hemodialysis refers to how well toxins and waste products are removed from the patient's blood and has a major impact on their well-being. Dialysis delivery should be adequate to improve adequacy of life and to prolong survival [10]. Studies have indicated an increase in morbidity and mortality among patients with inadequate dialysis [11].

The urea removal indexes help to calculate the adequacy of hemodialysis. The urea removal indexes include urea reduction ratio (URR), single-pool (sp*Kt*/*V*), equilibrated (e*Kt*/*V*), and the weekly standard index (std *Kt*/*V*). This study used URR and sp*Kt*/*V* to calculate hemodialysis adequacy.

### 1.1. Urea Reduction Ratio (URR) Index

The URR can be assessed by measuring the blood urea nitrogen (BUN) level before and after dialysis (8). It is calculated as follows:

(1) $$\begin{array}{c} \text{URR}=\frac{\left(\text{predialysis BUN}-\text{postdialysis BUN}\right)}{\left(\text{predialysis BUN}\right)\times 100\%}. \end{array}$$

### 1.2. Single-Pool Index (sp*Kt*/*V*)

The sp*Kt*/*V* index is defined as the amount of serum that is cleared from urea via the distribution volume, in relation to the urea reduction ratio during hemodialysis. Parameter K is the dialyzer blood water urea clearance that is provided by the filter (measured as liters per hour), *t* is the duration of the hemodialysis session in hours, and *V* is the volume of urea distribution in combination with the body water in liters.

The parameters sp*Kt*/*V* and URR are connected mathematically as follows:

(2) $$\begin{array}{c} \frac{\text{sp}Kt}{V}=-\ln\left(1-\text{URR}\right), \end{array}$$

where ln stands for natural logarithm.

In addition, the sp*Kt*/*V* index counts the ultrafiltration and the urea production. However, none of the parameters surpass the other as a denouement criterion (9–12).

The National Kidney Foundation-Kidney Disease Outcomes Quality Initiative (NKF-KDOQI) 2006 guidelines recommend sp*Kt*/*V* > 1.2 or URR ≥ 65% for maintenance hemodialysis, when a three times per week hemodialysis program is applied. These values constitute the lowest possible values and not the target values, which are 1.4 for sp*Kt*/*V* and 70% for URR.

No previous studies have been done to determine the adequacy of hemodialysis in Dar es Salaam. Since the cost of hemodialysis is high and entails significant patient time, it is important that hemodialysis should be done adequately. There are approximately 300 patients who are undergoing hemodialysis in Dar es Salaam.

This study was aimed at determining the adequacy of hemodialysis and its associated factors in patients undergoing chronic hemodialysis in Dar es Salaam. Findings from this study will provide important feedback information to the dialysis centers in Dar es Salaam which can serve to improve dialysis services in the region. Furthermore, this study built awareness among the dialysis centers who were not already measuring the adequacy of hemodialysis for their patients.

## 2. Materials and Methods

This was a cross-sectional study carried out in four dialysis centers in Dar es Salaam, which included Muhimbili National Hospital, TMJ Hospital, Aga Khan Hospital, and Tumaini Hospital. The finite sample size was 143. Data were collected over a period of seven months. The study population included all consenting adults ≥18 years of age undergoing chronic hemodialysis who were in a steady state at the time of data collection. All patients had an average dialysate flow rate of 500 ml/min.

The measurement of blood urea before and after dialysis was done for previous month in the first week of the next month. The calculation of URR and sp*Kt*/*V* was done using the formulas described above. Relevant data on sociodemographics and treatment characteristics were collected using structured questionnaires and patient's hospital records.

Urea after dialysis was taken following KDOQI guidelines as follows:

1. The ultrafiltration rate was set to zero.
2. The blood pump was slowed to 100 ml per minute for 10–20 seconds.
3. The pump was then stopped.
4. A sample was drawn, either from arterial blood line sampling port or from the tubing attached to arterial needle.

Data were analyzed using SPSS 23. Relevant frequencies and appropriate tables were generated for different variables. Means and proportions were calculated for appropriate variables. All associated factors for inadequate dialysis were analyzed using Chi-squared test. A logistic regression analysis was done to find out independent associating factors for inadequate hemodialysis. Statistical significance was set at *p* value < 0.05.

Permission to conduct the study was sought from relevant ethical committees at MNH and Muhimbili University of Health and Allied Sciences (MUHAS) (Ref no. MU/PGS/SAEC/Vol. XIV). Permission from the dialysis centers was also sought.

All patients were entered into the study after an informed consent, either given by the patients themselves or their guardians in the case where patients were not able to. The data obtained during the study were kept anonymous.

## 3. Results

### 3.1. Social Demographic and Clinical Characteristics (Table 1)

A total of 143 participants were enrolled in the study. About two-thirds (65.7%) were males. The mean age (±SD) was 51.7 ± 1.2 years.

### 3.2. Proportion of Patients Receiving Inadequate Hemodialysis

The mean URR and *Kt*/*V* were 60.9 ± 12.0% and 1.1 ± 0.3, respectively. The proportion of patients receiving adequate hemodialysis among patients undergoing chronic hemodialysis in Dar es Salaam based on URR was 34.3% and based on *Kt*/*V* was 40.6%.

By *Kt*/*V* criteria, males had significantly a higher prevalence of inadequate hemodialysis (65.6% versus 48.0%, *p*=0.048) but not by URR criteria (70.2% versus 57.1%, *p*=0.139)(Table 2).

Patients using a dialyzer surface area of less than 1.4 m^2^ had significantly more inadequate hemodialysis as compared to those with dialyzer surface area ≥1.4 m^2^ (69.0% versus 41.2%, *p*=0.023, by URR criteria) (62.7% versus 35.3%, *p*=0.033, by *Kt*/*V* criteria) (Table 3).

By *Kt*/*V* criteria, patients who had hemoglobin of <10 g/dl in the last month had significantly more inadequate hemodialysis as compared to those who had hemoglobin of ≥10 g/dl. This association was not seen when using URR criteria (71.4% versus 61.3%, *p*=0.203, by URR criteria) (69.8 versus 51.3%, *p*=0.029, by *Kt*/*V* criteria) (Table 3).

The logistic regression analysis was done to find independent association of factors with inadequate hemodialysis in patients undergoing chronic hemodialysis in Dar es Salaam. Factors that showed association in univariate analysis were included in the regression model. None of the factors showed independent association with inadequate hemodialysis in patients undergoing chronic hemodialysis by both URR and *Kt*/*V* (Table 4).

## 4. Discussion

This was a cross-sectional study looking at the prevalence of inadequate dialysis and its associated factors.

In this study, the mean *Kt*/*V* was 1.1 and URR was 60.9%. This indicated that generally patients achieve less than the hemodialysis target therapy as per NKF-KDOQI 2006 recommendations [16]. These values were however better than the values reported in Iran (*Kt*/*V* = 0.93, 1.17 and URR = 53%) [17, 18]. This study revealed similar findings to those carried out in other developing countries such as Brazil, Nigeria, Nepal, and Pakistan [19–23] but they differed from those reported from developed countries such as the United States [24] and from other five European countries as part of the DOPPS study where the mean delivered *Kt*/*V* varied from 1.28 to 1.50 [25].

In this study, it was found that about one-third (34.3%) and 40.6% receive target URR (≥65%) and *Kt*/*V* (>1.2), respectively. Similar values for URR and *Kt*/*V* were found in studies done in Sari [26] and Rasht states and in Qom [27, 28]. A study in Ardabil showed that only 10% of patients received adequate hemodialysis [29]. This was in contrast to studies done in developed countries (DOPPS study and in the United States) where URR ranged from 60–90% and *Kt*/*V* was more than 1.2 [24, 25]. The substantial discrepancy in hemodialysis adequacy between developed and developing regions may be resulting from the more frequent use of high-flux dialysis and higher blood flow rates in the developed countries than in the developing regions like Dar es Salaam (19–60% versus 0.0%, and 251–322 ml/min versus 269 ml/min, respectively) [24, 25]. Some centers in Dar es Salaam do not adequately follow the KDOQI guidelines and this could have resulted in the lower proportion of adequate hemodialysis.

This study found three factors that were significantly associated with inadequate dialysis in the univariate analysis.

Males were more likely to have inadequate hemodialysis than females. These results were similar to a study done in Iran where they found that males had three times more inadequate hemodialysis as compared to females [17]. Since the dialysis efficiency is inversely proportional to the urea distribution volume (*V*), it is expected that patients with higher values of height will have less dialysis efficiency and this may at least in part explain the difference as men are generally taller than women. It may also be due to less muscular mass, less body activity, and better diet observance among females [28].

This study also found that patients using hemodialyzers with surface area less than 1.4 m^2^ received significantly less hemodialysis dose than those who used dialyzers with surface area of more than or equal to 1.4 m^2^. Hemodialyzers with smaller surface area are known to give less adequate hemodialysis than those having larger surface area. This was shown in a study done in Bangladesh where they found that increasing the surface area of a dialyzer membrane (from 1.2 to 1.3 m^2^) increased the adequacy of hemodialysis by 10.4% (by URR criteria) and 19.7% (by *Kt*/*V* criteria) [30]. Similarly, Panagoutsos et al. showed that increasing the surface area of the dialyzer membrane from 1.15 m^2^ ± 0.1 to 1.7 m^2^ increased the *Kt*/*V* from 0.93 ± 0.19 to 1.55 ± 2.9 (*p* < 0.05) and URR from 52 ± 8% to 71 ± 7% (*p* < 0.05); that is, there was 66.7% and 36% increment in *Kt*/*V* and URR, respectively [31].

This study also found that patients with hemoglobin of less than 10 g/dl had more inadequate hemodialysis as compared to those who had hemoglobin of 10 g/dl. A study in South Africa showed that increasing the dose of hemodialysis from a *Kt*/*V* of 0.8 to 2 increased the hemoglobin level from 8.5 g/dl to more than 10 g/dl [32]. The inadequate urea clearance by hemodialysis can result in malnutrition, anemia, and functional impairment with an increased risk of hospitalization, morbidity, and mortality. Anemia could also be a marker for inflammation and malnutrition, which can influence *Kt*/*V*, but this was not evaluated in this study.

The logistic regression analysis failed to show any significant association between the above factors with hemodialysis adequacy. This probably indicates that hemodialysis adequacy is not dependent on one variable alone, but rather it is a function of different factors.

Strengths of this study were that it was a multicenter study which enabled participants from different backgrounds to be included. Many factors were evaluated for them associated with hemodialysis adequacy. Furthermore, hemodialysis adequacy was assessed using both *Kt*/*V* and URR.

The study had a few limitations. Firstly, the investigations for different patients were done at their respective centers which may have brought some variability in the results. Data on some confounding factors for *Kt*/*V* were not collected in this study such as albumin, inflammatory markers (CRP and ferritin), residual renal function, and other comorbidities unrelated to CKD.

## 5. Conclusion

Hemodialysis inadequacy is frequent in Dar es Salaam and is associated with male gender, dialyzer surface area, and hemoglobin level. Giving the correct hemodialysis dose could help improve hemodialysis adequacy.

## Figures and Tables

**Table 1:** Sociodemographic characteristics of the study population.

Characteristics	Frequency (*n* = 143)	Percent (%)
Age (years)		
<40	27	18.9
≥40	116	81.1
Sex		
Male	94	65.7
Female	49	34.3
Marital status		
Married	102	71.3
Widowed	14	9.8
Divorced	9	6.3
Single	18	12.6
Education level		
No formal education	5	3.5
Primary	33	23.1
Secondary	62	43.3
College/University	43	30.1
Body mass index (kg/m^2^)		
Underweight (<18.5)	14	9.8
Normal (18.5–24.9)	81	56.6
Overweight (25–29.9)	40	28.0
Obese (>30)	8	5.6

**Table 2:** Demographic and clinical factors associated with inadequate hemodialysis.

Factors	URR		*p* value	*Kt*/*V*		*p* value
	Inadequate (%)	Adequate (%)		Inadequate (%)	Adequate (%)	
Age (years)						
<40	19 (70.4)	8 (29.6)	0.657	15 (55.6)	12 (44.4)	0.435
≥40	75 (64.7)	41 (35.3)		69 (59.5)	47 (40.5)	
Sex						
Male	66 (70.2)	28 (29.8)	0.139	61 (65.6)	32 (34.4)	**0.048**
Female	28 (57.1)	21 (42.9)		24 (48.0)	26 (52.0)	
Education level						
No formal education	3 (60.0)	2 (40.0)	0.741	3 (60.0)	2 (40.0)	0.957
Primary	21 (63.6)	12 (36.4)		20 (58.8)	14 (41.2)	
Secondary	39 (62.8)	23 (37.2)		35 (57.4)	26 (42.6)	
College/University	31 (72.1)	12 (27.9)		27 (62.8)	16 (37.2)	
Body mass index (kg/m^2^)						
Underweight (<18.5)	10 (71.4)	4 (28.6)	0.932	10 (66.7)	5 (33.3)	0.963
Normal (18.5–24.9)	54 (66.7)	27 (33.3)		48 (59.3)	33 (40.7)	
Overweight (25–29.9)	25 (62.5)	15 (37.5)		22 (56.4)	17 (43.6)	
Obese (>30)	5 (62.5)	3 (37.5)		5 (62.5)	3 (37.5)	
Underlying disease						
Hypertension alone	54 (64.3)	30 (35.7)	0.381	48 (57.1)	36 (42.9)	0.832
Diabetes alone	11 (57.9)	8 (42.1)		10 (55.6)	8 (44.4)	
Hypertension and diabetes	20 (66.7)	10 (33.3)		19 (63.3)	11 (36.7)	
Others	9 (90.0)	1 (10.0)		7 (70.0)	3 (30.0)	

**Table 3:** Treatment characteristics associated with inadequate hemodialysis.

Factors	URR		*p* value	*Kt*/*V*		*p* value
	Inadequate (%)	Adequate (%)		Inadequate (%)	Adequate (%)	
Dialyzer surface area (m^2^)						
<1.4	87 (69.0)	39 (31.0)	**0.023**	79 (62.7)	12 (31.6)	**0.033**
≥1.4	7 (41.2)	10 (58.8)		6 (35.3)	46 (43.8)	
Vascular access in the last month^*∗*^						
Temporary	27 (71.1)	11 (28.9)	0.420	26 (68.4)	12 (31.6)	0.226
Permanent	67 (63.8)	38 (36.2)		59 (56.2)	46 (43.8)	
Months since dialysis initiation						
3–12	53 (63.1)	31 (36.9)	0.428	48 (57.1)	36 (42.9)	0.467
>12	41 (69.5)	18 (30.5)		37 (62.7)	22 (37.3)	
Number of dialysis sessions per week						
1	0	2 (100.0)	0.128	0	2 (100.0)	0.222
2	15 (62.5)	9 (37.5)		15 (62.5)	9 (37.5)	
3	79 (67.5)	38 (32.5)		70 (59.8)	47 (40.2)	
Dialysis sessions in a month						
2–11	32 (60.4)	21 (39.6)	0.300	31 (58.5)	22 (41.5)	0.901
>11	62 (68.9)	28 (31.1)		54 (60.0)	36 (40.0)	
Blood flow rate (ml/min)						
<250	13 (65.0)	7 (35.0)	0.941	13 (65.0)	7 (35.0)	0.566
≥250	81 (65.9)	42 (34.1)		72 (58.5)	51 (41.5)	
Ultrafiltration (liters)						
0–2	65 (65.7)	34 (34.3)	1.000	60 (60.6)	39 (39.4)	0.716
2–4	29 (65.9)	15 (34.1)		25 (56.8)	19 (43.2)	
Hemoglobin (g/dL)						
<10	45 (71.4)	18 (28.6)	0.203	44 (69.8)	19 (30.2)	**0.029**
≥10	49 (61.3)	31 (38.7)		41 (51.3)	39 (48.7)	

^*∗*^Temporary access included central venous catheters and permanent access included arteriovenous fistulae and arteriovenous graft in 3 patients.

**Table 4:** Logistic regression analysis for factors with inadequate hemodialysis.

Characteristic	Adjusted OR	95% CI	*p* value
By URR			
Sex—male	0.58	0.28–1.20	0.144
Hb < 10 g/dL^b^	0.67	0.33–1.38	0.278
DSA < 1.4 m^2c^	1.58	0.54–4.60	0.402
By *Kt*/*V*			
Sex—male^a^	0.49	0.24–1.00	0.050
Hb < 10 g/dL^b^	0.49	0.24–1.00	0.050
DSA < 1.4 m^2c^	1.23	0.41–3.72	0.708

a: versus females; b: versus Hb > 10 g/dL; c: versus DSA > 1.4 m^2^.

## Data Availability

The data that support the findings of this study are available from the Muhimbili University of Health and Sciences, but restrictions apply to the availability of these data, which were used under license for the current study, and so are not publicly available. Data are however available from the authors upon reasonable request and with permission from the Muhimbili University of Health and Allied Sciences.
